# Seasonal Mortality Patterns Analyzing Epidemiological Impact of COVID-19 on Overall Mortality Rates in Belgrade, Serbia Over Three-Year Period (2020–2023): Mental Health Consequences and Public Health Implications

**DOI:** 10.3390/jcm14103290

**Published:** 2025-05-08

**Authors:** Jelena Milic, Zoran Kokic, Predrag Kon, Milica Vucurovic, Sonja Novak, Nadezda Popovic, Vuk Marusic

**Affiliations:** 1 Institute of Public Health of Serbia “Dr. Milan Jovanovic Batut”, Dr. Subotica 5, 11000 Belgrade, Serbia; dr.milica.vucurovic@gmail.com; 2 European University KALLOS, Ratarski put 8a, 11000 Belgrade, Serbia; 3 Community Health Centre Voždovac, 11010 Belgrade, Serbia; 4 Belgrade City Institute of Public Health, 11108 Belgrade, Serbia; 5 Department of Epidemiology, Faculty of Medicine, University of Niš, 18000 Niš, Serbia; 6 University Clinical Center Niš, 18000 Niš, Serbia; 7 Institute of Epidemiology, Medical Faculty, University of Belgrade, 11000 Belgrade, Serbia; vuk.marusic@med.bg.ac.rs

**Keywords:** population health, health promoting community design, seasonal mortality, respiratory diseases, public health, epidemiology

## Abstract

**Background/Objectives**: Seasonal variations in mortality rates are well-documented, particularly during the winter months when mortality typically increases. This rise in mortality, ranging from 5% to 25%, is often associated with chronic cardiovascular and respiratory diseases. Understanding these seasonal fluctuations is essential for guiding public health interventions. This study analyzes mortality rates and excess mortality in Belgrade from March 2020 to May 2023, focusing on the impact of the COVID-19 pandemic on overall mortality trends. The primary objective of this study is to assess the impact of the COVID-19 pandemic on mortality rates in Belgrade during the study period. The first secondary objective is to evaluate seasonal variations in mortality, with a focus on the 10.57% overall increase in mortality, and to highlight the 34.23% rise in winter mortality recorded in 2020. The second secondary objective is to assess the effectiveness of public health measures in mitigating excess mortality during this period. **Methods**: A descriptive epidemiological approach was used to analyze monthly mortality data from the City Bureau of Statistics. Mortality rates were standardized using direct standardization and compared winter (December-February) and non-winter (March-November) periods. Trends, percentage increases, and age-specific mortality were analyzed based on the 2011 census methodology. **Results**: Mortality rates in Belgrade ranged from 1115.67 to 1267.19 deaths per 100,000 inhabitants, with an average of 1205.62. Standardized mortality rates ranged from 936.49 to 1111.67, averaging 1021.64. The winter months showed higher mortality, averaging 1716 deaths per 100,000, compared to 1558 in the non-winter months. **Conclusions**: The winter months exhibited significantly higher mortality rates, likely exacerbated by the COVID-19 pandemic. Targeted public health policies and interventions are necessary to reduce seasonal mortality risks during future public health crises.

## 1. Introduction

### 1.1. Purpose

Understanding mortality rates and excess mortality is essential for assessing public health impacts. Mortality rates reflect population health and can signal the effects of epidemics, socioeconomic factors, and environmental conditions. The COVID-19 pandemic, caused by SARS-CoV-2, has profoundly altered global mortality patterns, exacerbated health disparities, and revealed healthcare system vulnerabilities. This study analyzes mortality rates and excess mortality in Belgrade from March 2020 to May 2023, focusing on the pandemic’s impact on Serbia’s overall mortality.

#### 1.1.1. Historical Trends in Mortality Rates

Serbia, an upper-middle-income nation in Southeastern Europe on the Balkan Peninsula, has endured significant economic and healthcare challenges over the past three decades. These struggles were amplified by the breakdown of the last vestiges of Yugoslavia and the resulting regional conflicts, which severely weakened the country’s healthcare infrastructure and overall stability. During this period, the combination of social unrest, deteriorating living standards, and a lack of essential resources led to a marked decline in public health. The disarray within the healthcare system, coupled with shortages of medical supplies and poor nutrition, exacerbated the situation, contributing to a notable increase in mortality rates [1].

By 2014, cardiovascular diseases (CVD) were accountable for over half of all deaths in Serbia. In the early 2010s, the country recorded particularly high standardized mortality rates for CVD—991 per 100,000 men and 836 per 100,000 women in 2013. These figures reflect a broader trend from the preceding decades, where the rise in deaths linked to cardiovascular diseases, respiratory disorders, and mental health issues became more pronounced. This was largely due to the increasingly limited access to healthcare services. The repercussions of these challenges are still felt in Serbia’s health system today and highlight the long-term impact of prolonged instability [1].

Mortality rates in Serbia have fluctuated significantly over the past century and are shaped by various social, economic, and environmental factors. During periods of socioeconomic crises, such as the financial instability of the 1990s and early 2000s, mortality rates saw notable changes. A study examining the period between 1987 and 1999 demonstrated that socioeconomic turmoil, particularly the effects of war and economic decline, significantly influenced mortality trends. The research highlighted a clear shift in mortality rates during this time, underscoring the impact of the broader socio-political crisis on public health in Serbia. These findings emphasize how major social upheavals can drive fluctuations in mortality rates, which specifically reflect the consequences of such crises [2]. This historical backdrop demonstrates how external factors, including political turmoil and economic challenges, can contribute to shifting mortality patterns, thus highlighting the need for a resilient healthcare system and policies that can mitigate the impact of such crises on public health [3].

#### 1.1.2. Impact of Socioeconomic Disparities

Socioeconomic disparities have long been a critical factor influencing mortality rates in Serbia, with significant differences between urban and rural populations. Studies consistently show that individuals in lower socioeconomic groups experience higher mortality rates driven by factors such as limited access to healthcare, poor nutrition, and higher rates of chronic diseases. In Serbia, these disparities are particularly evident in Belgrade, where a complex socioeconomic landscape means that the wealthiest citizens have better access to healthcare services, while the most vulnerable populations struggle with inadequate resources [4].

People living in lower-income areas of Belgrade, despite being in an urban environment with theoretically better access to healthcare, often faced barriers such as underfunded healthcare facilities and overcrowded hospitals, which reduced the effectiveness of treatments. The COVID-19 pandemic only deepened these existing inequalities as the virus disproportionately affected older adults and those with pre-existing conditions, many of whom belong to the most disadvantaged socioeconomic groups. Understanding how these socioeconomic factors shape mortality trends is essential for developing targeted public health interventions that address the underlying causes of mortality rather than just its symptoms.

#### 1.1.3. Regional Studies and Seasonal Variations

Regional studies conducted in Serbia, particularly in Belgrade and its surrounding areas, reveal that seasonal variations in mortality are not only a global phenomenon but are also deeply ingrained in the local context. Mortality rates in Belgrade typically rise during the winter months, with some studies showing an increase of up to 25% during this period; this is primarily due to cardiovascular diseases, respiratory conditions, and influenza-related deaths [5]. One of the key factors contributing to these seasonal variations is the aging population in Belgrade, which is more susceptible to cold weather and respiratory infections. The winter months in the city bring not only low temperatures but also significant air pollution, which further exacerbates health risks, especially for individuals with pre-existing cardiovascular or respiratory conditions [6]. A study by the Institute of Public Health of Serbia in 2019 identified that despite urban areas generally having better healthcare infrastructure, the concentration of pollution and the high number of elderly residents made Belgrade particularly vulnerable to the negative effects of winter. Understanding these seasonal trends is crucial for public health policy, as it can help identify high-risk periods and enable authorities to prepare for more effective responses such as increasing outreach programs for the elderly or improving air quality standards during the winter months [7].

#### 1.1.4. Role of Healthcare and Public Health Policy

The role of healthcare infrastructure and public health policies in shaping mortality trends is especially significant during public health crises, as demonstrated by the COVID-19 pandemic [8]. During the pandemic, Serbia’s healthcare system was severely strained, leading to inadequate care for non-COVID-related illnesses. The healthcare system, already under pressure due to years of underfunding and staff shortages, was further overwhelmed by the surge in COVID-19 cases, resulting in delayed diagnoses and treatment for other conditions, which contributed to excess mortality. Furthermore, the pandemic highlighted gaps in healthcare access, particularly for rural populations, which faced even greater barriers in seeking care due to geographical distances, lack of medical professionals, and limited healthcare resources [9]. The experience of the pandemic underscored the critical need for healthcare system reforms that improve capacity, accessibility, and quality of care, especially in underserved areas. Strengthening public health policies, such as improving the preparedness of healthcare systems for emergencies and ensuring that vulnerable populations are prioritized, will be essential for mitigating the impact of future health crises and reducing excess mortality [10].

### 1.2. Seasonal Variations in Mortality

Mortality rates exhibit seasonal fluctuations, especially in winter, where mortality can increase by 5% to 25%, mainly due to cardiovascular and respiratory conditions [11]. Cold weather, respiratory infections, and healthcare access are key factors contributing to these variations [12]. Research shows that winter exacerbates heart attacks, strokes, and respiratory diseases, leading to higher mortality rates [13]. In Belgrade, a city with harsh winters and an aging population, understanding seasonal mortality is crucial for effective public health responses [5]. Studies have consistently shown increased winter mortality, highlighting the need for policies to protect vulnerable groups [14].

### 1.3. Impact of COVID-19 on Mortality

The COVID-19 pandemic dramatically altered mortality rates in Serbia. The first case of COVID-19 in Serbia was reported on 6th March 2020. Since then, Serbia has registered several epidemic peaks, which have led to a considerable increase in premature mortality [15]. Beyond direct fatalities, a global phenomenon observed was the strain on healthcare services during the pandemic, which led to excess mortality from other conditions in Serbia as well as in other countries [16]. Excess mortality, defined as deaths exceeding the expected number, provides a broader measure of the pandemic’s impact [17]. A study found that excess mortality in Serbia was significantly higher than the reported COVID-19 deaths, thus underscoring the pandemic’s far-reaching effects on public health [18].

### 1.4. Objectives

Objectives: The primary objective of this study is to assess the impact of the COVID-19 pandemic on mortality rates in Belgrade during the study period. The first secondary objective is to evaluate seasonal variations in mortality, with a focus on the 10.57% overall increase in mortality, and to highlight the 34.23% rise in winter mortality recorded in 2020. The second secondary objective is to assess the effectiveness of public health measures in mitigating excess mortality during this period.

The findings are essential for guiding future health policies aimed at mitigating the effects of similar public health crises. Understanding seasonal variations and the specific impacts of the pandemic will help us develop targeted interventions and improve public health outcomes. Further research is needed to explore the causes of high mortality, especially among vulnerable groups, to better prepare for future health emergencies.

## 2. Materials and Methods

### 2.1. Study Design

This study employed a comprehensive descriptive epidemiological design to analyze mortality rates and assess excess mortality in Belgrade from March 2020 to May 2023.

### 2.2. Sample

Data for the study were sourced from two key institutions: the Republic Institute for Public Health of Serbia, which provided morbidity statistics, and the Republic Institute for Informatics and Statistics, from which mortality data were obtained. The combined dataset from these sources ensured a basis for the analysis.

### 2.3. Measures

The primary measures used in this study included the actual and expected mortality rates. The actual mortality rate was calculated based on the number of deaths observed within the study period, while the expected mortality rate was estimated using historical data dating back to 1995, adjusted for seasonal fluctuations. The proportional excess mortality rate (p-Excess MT) was the key metric for assessing excess mortality, representing the difference between observed and expected mortality rates.

### 2.4. Analysis

Statistical analysis was conducted using SPSS 19 i software. Linear regression was applied to identify trends in mortality rates over time. To model the expected mortality rates, we used Exponential Smoothing with a simple seasonal component. This approach accounted for seasonal fluctuations, enhancing the accuracy of the expected mortality estimates.

To calculate excess mortality, we first determined the actual mortality rate (observed deaths) and the expected mortality rate (derived from historical trends and seasonal adjustments). The difference between the actual and expected rates was computed to evaluate whether mortality was higher or lower than anticipated.

Finally, the difference between the actual and expected mortality rates was expressed as a proportion of the expected rate. This proportional difference was converted into a percentage to quantify excess mortality. A positive value indicated that mortality exceeded expectations, potentially reflecting the impact of adverse factors or interventions, while a negative value suggested that mortality was lower than expected, which could indicate favorable conditions or outcomes.

## 3. Results

**Figure 1:**
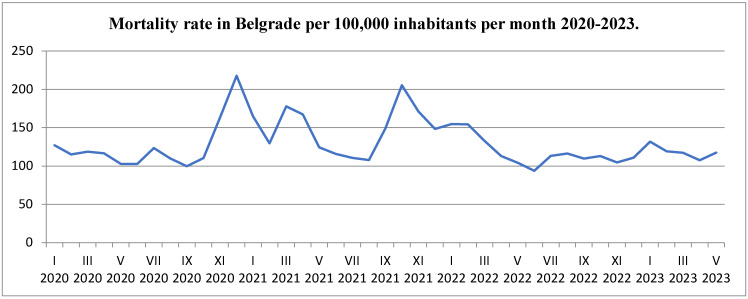
Mortality rate in Belgrade per 100,000 inhabitants per month 2020–2023.

During the study period from March 2020 to May 2023, we observed notable fluctuations in mortality rates in Belgrade (Figure 1). The lowest recorded mortality rate occurred in June 2022, with a value of 93.75 deaths per 100,000 inhabitants, corresponding to a total of 1579 deaths. Conversely, the highest mortality rate was documented in December 2020, when the rate surged to 217.65 deaths per 100,000 inhabitants, accounting for a total of 3688 deaths.

**Figure 2:**
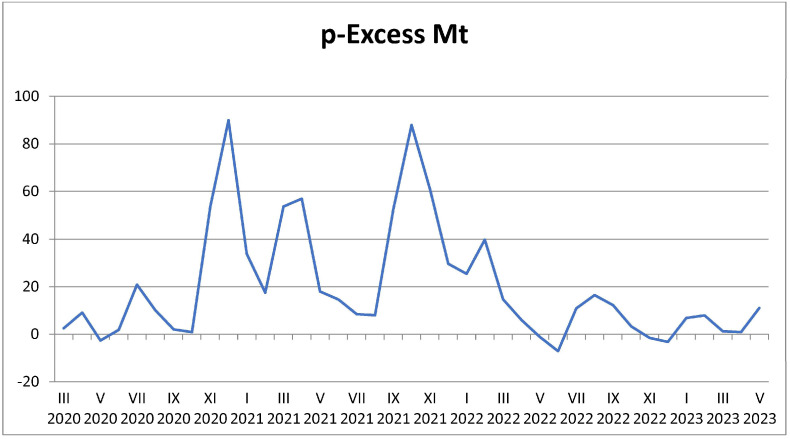
p-Excess Mt.

In addition to analyzing overall mortality rates, we specifically examined the proportional excess mortality rate (p-Excess MT) (Figure 2) as a key indicator of the impact of various conditions and events during this period. The lowest value of p-Excess MT was recorded in June 2022 at −7.25, indicating a slight decrease in mortality compared to what was expected based on historical data. This negative value suggests that mortality during this month was lower than anticipated, potentially reflecting the positive effects of public health interventions or a return to baseline health conditions following periods of higher mortality.

The decrease in mortality in June 2022 is specifically attributed to the successful implementation of public health interventions, such as vaccination campaigns and the gradual lifting of lockdowns, which likely contributed to the reduction of excess mortality during this period.

This figure depicts the monthly mortality rates per 100,000 inhabitants in Belgrade from 2020 to 2023, offering a clear representation of mortality trends over the course of the COVID-19 pandemic. The data are presented as a time series, allowing for a visual comparison of fluctuations in mortality on a month-by-month basis:

- Mortality Rate (per 100,000): The y-axis of the figure shows the number of deaths per 100,000 people, while the x-axis represents each month from March 2020 to May 2023. The data show the overall trend in mortality rates during this period, with certain months exhibiting notable increases, particularly during the winter months and during peaks of COVID-19 infection.
- Peak Mortality Months: Significant peaks in the graph correlate with the height of the COVID-19 pandemic in Belgrade. These peaks reflect the sharp increase in mortality during high infection rates, highlighting the pandemic’s profound impact on public health.

The peak mortality months are also linked to specific public health measures such as lockdowns, social distancing, and the timing of vaccination rollouts, which played a crucial role in mitigating excess mortality during these months.

This figure emphasizes the direct relationship between the COVID-19 pandemic and increased mortality rates, particularly in months marked by high infection rates. By showing these trends, the figure helps illustrate the scale of excess mortality and provides evidence for the need for effective public health interventions, particularly during periods of viral outbreaks and seasonal variations in mortality.

This figure illustrates the *p*-value of excess mortality, which is a statistical representation used to assess whether the mortality rates during the pandemic period were significantly higher than expected based on historical data. Excess mortality is defined as the number of deaths above and beyond the normal mortality rate for a specific time period, which can be influenced by various factors, including the COVID-19 pandemic.

p-Excess Mortality (y-axis): The y-axis represents the *p*-value, a measure of statistical significance, indicating whether the observed excess mortality is significantly higher than what would be expected in a typical year. The *p*-value helps assess the reliability of the observed increase in mortality, with lower *p*-values indicating a stronger likelihood that the observed excess mortality is not due to random chance.

Temporal Variation (x-axis): The x-axis represents the time period from 2020 to 2023, broken down by months. The graph shows how excess mortality varied over time, with higher *p*-values indicating periods of more significant excess mortality, often coinciding with high infection rates and peak COVID-19 waves.

The highest p-Excess MT value was observed in December 2020, reaching 89.96. This substantial positive value indicates that mortality was significantly higher than expected during this month due to the overwhelming impact of COVID-19 on healthcare systems and vulnerable populations.

This marked increase in excess mortality is attributed to the combined effects of healthcare strain and the absence of early intervention strategies such as widespread vaccination or enhanced healthcare capacity.

This figure provides insight into the temporal and statistical significance of excess mortality during the pandemic, reinforcing the notion that the pandemic was a key factor in increased death rates. By analyzing *p*-values, the figure helps quantify the impact of COVID-19 on the overall mortality burden and highlights the need for targeted health interventions, particularly during periods of high excess mortality.

In stark contrast, the highest p-Excess MT value was observed in December 2020, reaching 89.96. This substantial positive value indicates that mortality was significantly higher than expected during this month. The elevated p-Excess MT highlights the adverse impact of various factors, which may include the onset of the COVID-19 pandemic, increased strain on healthcare resources, and other socioeconomic challenges that could have exacerbated health outcomes.

Overall, the findings illustrate a complex interplay between actual and expected mortality rates throughout the observed period, emphasizing the necessity for continuous monitoring and assessment of public health policies in response to changing conditions. The variation in mortality rates and p-Excess MT values underscores the ongoing challenges faced by healthcare systems and the importance of timely interventions to mitigate excess mortality.

From March 2020 to July 2022, the Republic of Serbia recorded a total of 2,029,400 cases of SARS-CoV-2 infection, alongside 293,373 deaths from all causes. The peak number of SARS-CoV-2 infections occurred in January 2022, with 378,459 cases reported. In terms of mortality, the highest number of deaths attributed to all causes during this period was recorded in December 2020, totaling 17,321 fatalities.

Utilizing linear regression analysis, we established that the rate of SARS-CoV-2 infection per 100,000 inhabitants is a statistically significant predictor of general mortality rates per 100,000 inhabitants. The regression analysis yielded an F-value of 18.75 with a *p*-value of less than 0.001, indicating a strong statistical significance. The model explains 41.9% of the variability in mortality, highlighting the relationship between SARS-CoV-2 infections and overall mortality in the population.

We further found that SARS-CoV-2 infection rates were significantly correlated with increased mortality, particularly during high infection periods. Public health interventions, such as vaccination campaigns and social distancing, are highlighted as essential tools in mitigating this mortality relationship.

Additionally, the analysis indicates that effective public health interventions led to a marked reduction in the slope of this relationship during periods of intervention, further confirming the importance of timely responses.

**Figure 3:**
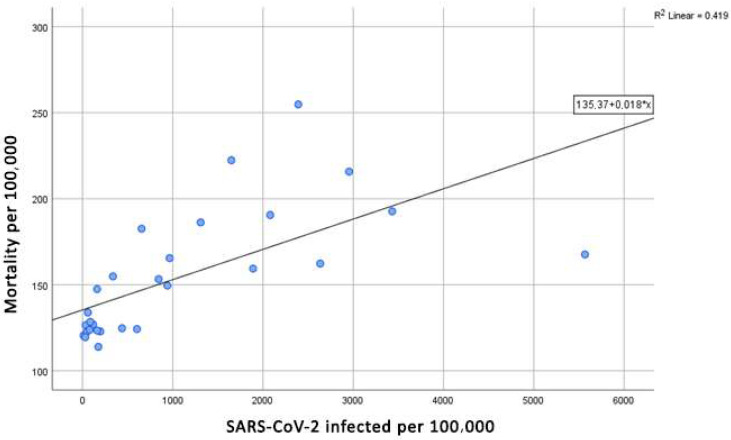
Mortality per 100,000 in regard to SARS-CoV-2 infected per 100,000.

The regression formula indicates that the mortality rate per 100,000 individuals can be expressed as a baseline value of 135.37, to which an additional component is added. This component increases the mortality rate by 0.018 for each case of SARS-CoV-2 infection per 100,000 individuals (Figure 3). This relationship illustrates that as the number of SARS-CoV-2 cases increases, so does the mortality rate, reflecting the disease’s impact on overall health outcomes.

This figure illustrates the relationship between general mortality rates (per 100,000 inhabitants) and the number of SARS-CoV-2 infections (per 100,000 inhabitants) in Belgrade from March 2020 to May 2023. The purpose of this figure is to visually capture the correlation between the progression of the COVID-19 pandemic and its direct impact on overall mortality trends:

- Mortality per 100,000: The first part of the figure shows the fluctuations in mortality rates, represented by the number of deaths per 100,000 inhabitants. These rates are plotted over time, allowing a visual comparison of periods of high and low mortality. The data highlight the sharp increases in mortality, particularly during waves of COVID-19 infection.
- SARS-CoV-2 Infected per 100,000: The second component of the figure tracks the number of confirmed SARS-CoV-2 cases per 100,000 individuals over the same period. Peaks in infection rates are visible, with notable surges corresponding to significant increases in mortality.

The relationship between these two variables now clearly links public health interventions to trends in both SARS-CoV-2 infection rates and mortality rates. This highlights the critical role of controlling infection transmission to reduce excess mortality.

The visual relationship between these two variables suggests that higher rates of SARS-CoV-2 infection are strongly associated with higher mortality rates, particularly during peak infection periods. This emphasizes the direct role that COVID-19 plays in shaping mortality trends during the pandemic, highlighting the severe consequences for public health. The data underscore the importance of timely public health interventions to manage both the infection rates and their associated mortality.

Furthermore, the regression coefficient of 0.018 suggests a clear pattern: for approximately every 55 cases of SARS-CoV-2 infection per 100,000 population in a given month, we can expect one additional death per 100,000 population during that same month. This finding underscores the significant burden of COVID-19 on public health and highlights the critical need for effective interventions to manage infection rates and mitigate mortality.

The analysis of mortality rates in relation to SARS-CoV-2 infections reinforces the urgent necessity for public health measures aimed at controlling infections, particularly during periods of heightened transmission.

Overall, these results emphasize the urgent necessity for public health measures aimed at controlling SARS-CoV-2 infections to reduce overall mortality rates, particularly during periods of heightened transmission. The statistical relationship demonstrated in this study provides important insights for policymakers and health authorities in developing strategies to safeguard public health in the face of ongoing challenges posed by the pandemic.

## 4. Discussion

**Figure 4:**
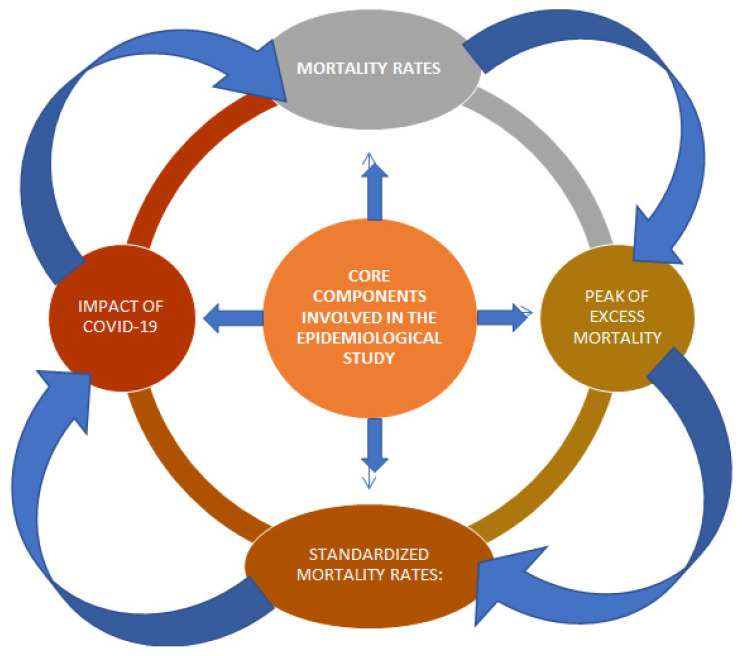
The core epidemiological components shaping the current study.

This study utilized a descriptive epidemiological approach to analyze mortality rates and excess mortality in Belgrade from March 2020 to May 2023 (Figure 4). According to the 2022 census, Belgrade’s population was 1.685 million. The lowest mortality rate occurred in June 2022 (93.76), while the highest was in December 2020 (217.65). Excess mortality peaked in December 2020 (89.96) and declined by June 2022 (−7.25). COVID-19 most affected individuals over 50 years old, with men suffering higher mortality rates than women.

The figure visually represents the key components of the epidemiological study conducted in Belgrade from March 2020 to May 2023. At the center of the figure is the primary focus of the study: Mortality and Excess Mortality Trends in Belgrade. Surrounding this central concept are four critical components: Mortality Rates, Excess Mortality, Standardized Mortality Rates, and Impact of COVID-19.

Each component is interrelated and helps in understanding the patterns of mortality data.

Mortality Rates: This section demonstrates monthly fluctuations, highlighting key periods of increased mortality, particularly during the winter months. These variations emphasize the seasonality of mortality in Belgrade, with notable peaks during the pandemic period.

Excess Mortality: Excess mortality is shown, particularly in December 2020, marking a significant increase during the COVID-19 pandemic. This field reflects the mortality beyond expected rates, illustrating the pandemic’s broader impact on public health.

Standardized Mortality Rates: This component offers a comparative framework by standardizing mortality rates across winter and non-winter periods. It highlights the seasonal variations in mortality and shows how these trends are affected by external factors, including the pandemic.

Impact of COVID-19: The figure also depicts the specific impact of COVID-19, particularly its contribution to age-specific mortality. It shows a higher mortality rate among individuals aged 50 and above, with a particular focus on men, reflecting the disease’s disproportionate effect on certain demographic groups.

Together, these components illustrate how seasonal variations, excess mortality, and the specific impacts of COVID-19 interact to shape overall mortality trends, providing a comprehensive view of the health consequences during the pandemic period.

### 4.1. COVID-19 Impact on Mortality Rates

**Figure 5:**
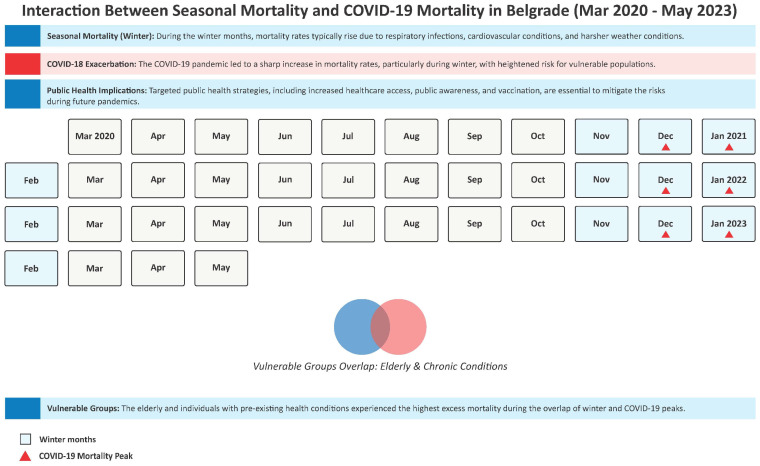
Interaction between seasonal mortality and COVID-19 mortality in Belgrade (March 2020–May 2023).

The findings align with earlier studies, such as the one from the United States where, by October 2020, COVID-19 had become the third leading cause of death for individuals aged 45–84 and the second for those over 85 years. This study corroborates the view that the elderly were particularly vulnerable to COVID-19 due to factors like pre-existing comorbidities [19]. Similarly, COVID-19 mortality in Serbia mirrored these patterns, with an increase in deaths starting in late 2020. In Figure 5, we present the interaction between seasonal mortality and COVID-19 mortality in Belgrade from March 2020 to May 2023 with relevant related factors of influence.

Figure 5 illustrates the complex interaction between seasonal mortality patterns and COVID-19 mortality in Belgrade from March 2020 to May 2023. The figure highlights the typical seasonal mortality trends in Belgrade, where mortality rates are observed to peak during the winter months, particularly from November to February, due to increased vulnerability to respiratory infections, cardiovascular diseases, and other seasonal conditions. These months are traditionally associated with higher mortality, especially among the elderly and individuals with chronic conditions. The red triangles on the graph mark the months during which COVID-19 cases peaked, showing the significant exacerbation of mortality during these periods. The overlap of the seasonal winter peaks and the COVID-19 surges reveals the compounded risk faced by vulnerable populations, such as the elderly and those with pre-existing chronic health conditions. This interaction led to a higher overall mortality rate during these specific months, with COVID-19 contributing to the seasonal increase in death rates.

The figure underscores the public health implications of these overlapping factors, emphasizing the heightened vulnerability of certain groups and the increased strain on healthcare systems. It also highlights the importance of targeted interventions during these critical months, such as improving access to healthcare, enhancing vaccination efforts, and focusing on chronic disease management, to mitigate the impacts of both seasonal and pandemic-related mortality.

### 4.2. Underreporting and Indirect Mortality

Official COVID-19 death counts are believed to underestimate the true toll due to underreporting, misclassification, and indirect deaths caused by healthcare system strain. Some estimates suggest COVID-19 deaths may be 50% higher than reported [20]. Furthermore, many individuals infected with SARS-CoV-2 but not tested are not counted, while others may have died from underlying conditions exacerbated by the pandemic. This underreporting, combined with non-COVID-19 deaths due to delayed treatments, points to a significant underestimation of the pandemic’s true mortality impact.

### 4.3. Excess Mortality and Broader Impacts

The broader impact of the pandemic is often measured by “excess deaths”, which capture the overall mortality during a given period, regardless of cause. Our study, following this approach, suggests that true excess deaths exceed official COVID-19 counts, encompassing indirect deaths as well [21]. In 2020, COVID-19 became the second leading cause of death in Belgrade, displacing diseases like ischemic heart diseases and cerebrovascular conditions [22]. This shift highlights the pandemic’s significant impact on public health, especially given that COVID-19 deaths affected a broader age range, including younger people with comorbidities.

### 4.4. Vulnerability of Middle-Aged Individuals

Our study refines earlier findings by showing that middle-aged men with comorbidities were most at risk of dying from COVID-19. Previous research has focused on elderly patients with pre-existing health conditions as the most vulnerable [23]. Our data suggest that premature mortality from COVID-19 was not limited to older adults but also affected individuals who would have otherwise had a longer life expectancy. These findings warrant further research using the Burden of Disease methodology to estimate the broader impact of the pandemic on life expectancy at both national and regional levels.

### 4.5. Non-Clinical Factors Influencing Mortality

In addition to clinical factors, non-clinical determinants such as socioeconomic status, healthcare access, governmental responses, and public health policies significantly shaped the pandemic’s impact. Income inequality, limited access to healthcare, and delayed treatment due to hospital overcrowding contributed to higher mortality rates [16,24,25,26,27,28,29,30,31,32,33]. Social and cultural factors also played a role. Public adherence to preventive measures like mask-wearing and social distancing influenced the trajectory of COVID-19 spread [34,35]. Additionally, economic and psychological factors, such as job loss and mental health challenges, exacerbated health disparities and increased vulnerability to the virus. The psychological toll, including stress and social isolation, may have also contributed to negative health outcomes, especially in those with underlying health conditions.

### 4.6. Seasonal Mortality and Psychological Consequences of Loss Leading to Grief and Complicated Grief

The COVID-19 pandemic has dramatically altered the landscape of mortality, not only due to the direct effects of the virus but also because of its profound psychological consequences [36]. Seasonal mortality, which tends to increase during the winter months due to heightened incidences of cardiovascular and respiratory diseases, was intensified by the pandemic. The cold weather, compounded by respiratory illnesses and a strained healthcare system, exacerbated mortality rates, particularly among vulnerable populations such as the elderly with pre-existing conditions [19]. This seasonal increase in deaths, coupled with the pandemic, created a unique public health crisis, heightening the psychological toll on individuals facing the loss of loved ones. Bereavement is widely recognized as one of the most traumatic life events. The loss of a loved one often leads to intense stress and increases the likelihood of developing depressive symptoms, major depressive episodes, anxiety-related disorders, impaired immune function, reduced quality of life, suicide, and higher mortality rates [37]. While most bereaved individuals will adjust within 6 to 12 months, experiencing a decrease in grief intensity and a return to a new, meaningful life without the deceased, some may struggle with prolonged or complicated grief [37]. For these individuals, adaptation may be delayed or prevented, leading to ongoing grief. Persistent grief is linked to functional impairment, sleep disturbances, high-risk behaviors, and an increased risk of cancer and cardiovascular diseases [37]. In community-dwellings, middle-aged and older adult persons with normal and complicated grief had both a shorter sleep duration and a lower sleep quality, which was mainly explained by depressive symptoms [38]. Also, a provisos study showed that [participants with complicated grief showed low levels of morning cortisol and low overall diurnal cortisol levels characteristic for a chronic stress reaction [39]. Typically, bereavement from seasonal mortality, which is one of the most frequent causes of death, creates similar levels of morbidity in cardiovascular and infectious diseases due to inflammation lowered resistance, leading to a cycle of distress and health decline that perpetuates itself over time, reinforcing a vicious spiral of seasonal impact.

One of the most significant emotional impacts of this crisis was the experience of anticipatory grief, especially among those with loved ones suffering in critical care or on respirators. Anticipatory grief occurs when individuals are confronted with the imminent loss of a loved one and begin mourning before the death happens [40]. This emotional burden is often complicated by feelings of helplessness, anxiety, and sorrow as individuals prepare for an expected loss. For families affected by COVID-19, this grief began early, as many watched loved ones struggle with the virus, often without the chance for meaningful goodbyes due to strict hospital protocols and isolation.

Once death occurred, many individuals were left to cope with complicated grief. The abrupt and unexpected nature of COVID-19-related deaths, coupled with the emotional isolation caused by lockdowns and restrictions, created a more intense grieving process. Complicated grief is characterized by prolonged and intense sorrow that interferes with daily life. This form of grief is often marked by difficulty accepting the loss, rumination on what could have been done differently, and a sense of being stuck in the mourning process. The lack of social support, rituals such as funerals, and the overall disruption of normal grieving practices made it difficult for many to process their emotions and begin the healing journey.

Additionally, the ongoing public health crisis made it harder to find closure, as individuals were deprived of traditional methods of grieving and honoring the deceased. This led to longer-lasting grief, as many individuals struggled to find a sense of emotional peace.

When combined with seasonal mortality trends, the pandemic’s impact on grief was particularly pronounced, leading to a dual crisis of both health and emotional well-being. Understanding how seasonal mortality and psychological grief intersect is essential for addressing the long-term mental health consequences of the pandemic. Providing adequate mental health support and acknowledging the unique challenges posed by these losses will be critical in helping individuals cope and recover.

### 4.7. Socio-Medical Perspective

From a socio-medical perspective, COVID-19’s impact on mortality underscores the importance of comprehensive public health and clinical measures. The premature mortality observed in Belgrade in 2020, despite the implementation of preventive measures, highlights the critical role of timely and effective interventions. Health promotion and adherence to preventive measures, such as vaccination, mask-wearing, and physical distancing, have been shown to reduce the spread of the virus and prevent deaths [41,42]. Our study suggests that the pandemic’s effects extend beyond direct clinical outcomes, with indirect mortality due to healthcare disruptions and socioeconomic factors. Addressing these broader determinants of health will be essential for future pandemic preparedness and mitigating the long-term consequences of COVID-19 on public health.

### 4.8. Insights on COVID-19 Mortality and Public Health

The COVID-19 pandemic significantly contributed to increased mortality in Belgrade. A recent study revealed that Registered COVID-19 deaths represented one-tenth of the total years of life lost (YLLs) in Serbia in 2020, with men contributing nearly twice as much to this total compared to women. On average, each registered COVID-19 death resulted in 12.32 years of life lost [15]. The findings emphasize the need for a multidisciplinary approach to understanding and addressing the pandemic’s complex impact on public health. While direct deaths from COVID-19 are critical, indirect deaths and non-clinical factors must also be considered in future mortality assessments. Additionally, timely and effective public health measures, such as vaccination campaigns and healthcare system preparedness, are essential in preventing further loss of life. These findings highlight the dual impact of COVID-19 on both public health and mental well-being.

### 4.9. Relevance for Local and Global Setting

In Belgrade, COVID-19 significantly worsened winter mortality, revealing the vulnerabilities in public health systems and underscoring the critical need for targeted healthcare interventions. The pandemic exacerbated the effects of seasonal illnesses, especially during the colder months when mortality rates are traditionally higher [43]. This highlighted the necessity of addressing both the direct and indirect impacts of winter health crises, which include not only viral infections but also underlying social determinants such as poverty, access to healthcare, and social isolation.

Globally, similar challenges have underscored the importance of seasonal preparedness, with many regions experiencing heightened mortality rates during the winter months due to COVID-19 and other respiratory diseases [44]. The pandemic has brought to the forefront the urgent need for equitable healthcare access, as populations in vulnerable conditions, such as the elderly and those with pre-existing health conditions, are disproportionately affected. Effective public health strategies must account for these factors and ensure that resources are distributed in a manner that addresses the needs of these at-risk groups.

Ultimately, the COVID-19 pandemic has served as a stark reminder that public health systems must be resilient and adaptive to seasonal fluctuations in mortality, emphasizing the need for comprehensive healthcare strategies that tackle both medical and social determinants of health. The pandemic has underscored the importance of addressing not only the direct health impacts of seasonal illnesses but also the broader societal factors, such as mental health, economic instability, and access to healthcare, that influence overall health outcomes. Public health systems must focus on enhancing their capacity to respond to both expected seasonal peaks in mortality and unexpected health emergencies. This includes providing accessible mental health support, ensuring that vulnerable populations have equitable access to healthcare, and strengthening healthcare infrastructure to handle surges in demand. Furthermore, addressing social determinants such as income, education, and housing is essential to improving long-term health outcomes. By taking a more holistic and proactive approach to health, public health systems can better protect populations from the adverse effects of seasonal mortality and be more resilient in the face of future health crises. The lessons learned from the COVID-19 pandemic should guide the development of more integrated and adaptable health strategies that can withstand both predictable and unforeseen health challenges.

### 4.10. Clinical Implications of COVID-19 Findings

COVID-19 research has shaped healthcare responses globally [45]. The virus spreads mainly through respiratory droplets, leading to preventive measures like mask-wearing and social distancing. Symptoms range from mild to severe, and some individuals remain asymptomatic, making control challenging [46]. Older adults and those with underlying conditions face higher risks, guiding healthcare priorities. Long-term effects, including respiratory, cardiovascular, and neurological issues, emphasize the need for post-recovery care. Research into antiviral drugs, immunotherapies, and vaccines has led to improved treatment protocols, enhancing care globally.

### 4.11. What Is Already Known on This Topic?

The COVID-19 pandemic has caused a significant rise in mortality rates globally, influenced by factors such as healthcare system strain, delayed treatments, and socioeconomic disparities. Public health strategies focusing on timely interventions, vaccination, and healthcare preparedness have been shown to reduce the impact of the pandemic on mortality. Table 1 gives a complete overview of the key aspects, findings, and implications of the current study.

### 4.12. What Does This Article Add?

This study specifically explores the long-term effects of COVID-19 on mortality in Belgrade, highlighting significant fluctuations in death rates and a peak of excess mortality in December 2020. It underscores the complex relationship between SARS-CoV-2 infections, healthcare challenges, and societal factors, providing valuable insights into the pandemic’s broader implications for public health.

### 4.13. What Are the Implications for Health Promotion Practice or Research?

The findings emphasize the need for targeted public health interventions, such as timely vaccination and addressing social determinants of health. Future research should focus on the long-term impacts of COVID-19, including “long COVID” and healthcare access, to reduce mortality disparities and inform future health crisis responses.

Future research on COVID-19 seasonal mortality and general seasonal mortality must be supported by high-quality evidence at the forefront of scientific inquiry. To guide evidence-based healthcare practices, guidelines, and policies, it is crucial to accurately identify, collect, and integrate all relevant evidence in a comprehensive, meaningful, and efficient manner. Approaches like systematic reviews and meta-analyses, when conducted with rigor, are essential tools for synthesizing and summarizing evidence on specific topics [47]. An important task in conducting a systematic review is reading the titles and abstracts of the retrieved references, which often number in the thousands, to determine which articles meet the predefined inclusion criteria. In the past, this process was performed by manually scanning through large stacks of printed titles and abstracts, followed by face-to-face meetings to discuss which references should be included. Today, the workflow of the review process is more streamlined with the use of computer software [48].

### 4.14. What Clinicians Seem to Be Missing: The Importance of Cardiovascular Diseases in COVID-19 Mortality

The relationship between cardiovascular disease and COVID-19 mortality has been extensively documented, with substantial evidence indicating that individuals with pre-existing cardiovascular conditions are at higher risk for severe COVID-19 outcomes, including hospitalization, intensive care unit admission, and death. Several studies, including the work of Ielapi et al. (2020) [49], have highlighted the role of cardiovascular disease as a biomarker for increased vulnerability to COVID-19 infection and worse prognosis. Ielapi et al. (2020) identified hypertension, coronary heart disease, heart failure, and arrhythmias as significant risk factors that correlate with increased mortality rates among COVID-19 patients. The pathophysiological mechanisms underlying this heightened risk include both direct viral injury to the cardiovascular system and indirect effects, such as the exacerbation of systemic inflammation and the alteration of coagulation parameters [49].

One key aspect of the elevated risk associated with cardiovascular disease is the impact of the SARS-CoV-2 virus on the angiotensin-converting enzyme 2 (ACE2) receptor, which is expressed in high concentrations in the heart and blood vessels. ACE2 is a crucial component in the regulation of the cardiovascular system, and its interaction with the virus may lead to direct myocardial injury, contributing to adverse outcomes in COVID-19 patients with pre-existing heart conditions. Ielapi et al. (2020) also discussed how patients with cardiovascular disease are more likely to exhibit elevated levels of ACE2 expression, which could increase their susceptibility to infection and worsen their prognosis. This finding underscores the importance of recognizing cardiovascular disease as a key factor in COVID-19 risk stratification [49].

Furthermore, the systemic inflammatory response triggered by COVID-19 infection, including the so-called “cytokine storm”, can exacerbate underlying cardiovascular conditions, such as atherosclerosis, and increase the likelihood of acute cardiac events, including myocardial infarction and arrhythmias. As noted by Ielapi et al. (2020), the inflammatory milieu in severe COVID-19 cases can trigger plaque rupture and lead to exacerbated cardiovascular damage. This is particularly concerning in patients with pre-existing coronary artery disease, where the combination of viral infection and inflammation may accelerate the progression of heart disease [49].

Despite the growing body of evidence linking cardiovascular disease with worse COVID-19 outcomes, it appears that clinicians have sometimes overlooked the importance of cardiovascular risk factors when assessing COVID-19 patients. Early in the pandemic, much of the clinical focus was placed on respiratory symptoms and the immediate effects of the virus on the lungs, while the cardiovascular implications were underemphasized. However, as research has evolved, it has become increasingly clear that cardiovascular health plays a central role in the severity and progression of COVID-19 infection [49].

In light of this, it is critical for clinicians to consider cardiovascular disease as a major factor in the management of COVID-19 patients. Early identification and intervention for those with underlying cardiovascular conditions can potentially mitigate the adverse effects of the virus. Furthermore, the integration of cardiovascular risk assessments into COVID-19 treatment protocols may improve patient outcomes and reduce mortality [49].

In summary, cardiovascular disease is a key determinant of COVID-19 severity and mortality. The work of Ielapi et al. (2020) and others has shown that clinicians must recognize the increased vulnerability of patients with cardiovascular conditions, particularly in the context of a pandemic where the interplay of viral infection, inflammation, and cardiovascular dysfunction can result in dire outcomes. A more comprehensive understanding of this relationship is essential for improving clinical practice and ultimately reducing COVID-19-related mortality, especially among individuals with pre-existing heart disease [49].

### 4.15. Study Limitations

While our study provides valuable insights into the impact of the COVID-19 pandemic on mortality rates in Belgrade, there are several limitations that must be acknowledged. First, while the concept of increased winter mortality and excess mortality during pandemics is well-documented, this study focuses specifically on the period from March 2020 to May 2023 in Belgrade, Serbia, and may not fully capture the broader patterns observed in other regions or countries. The findings may be influenced by local factors such as healthcare infrastructure, regional responses to the pandemic, and specific demographic characteristics that limit the generalizability of the results to other settings.

Additionally, although we utilized a rigorous statistical methodology and local data, the study’s emphasis on the Serbian context and the seasonal variations in mortality may not represent entirely novel findings. It is well-established that the winter months generally experience higher mortality due to factors such as respiratory infections and cardiovascular conditions, and the exacerbation of these trends during COVID-19 has been observed globally. Therefore, while the study provides important context-specific insights, the findings related to increased winter mortality and excess mortality may not present entirely new patterns. Rather, our research aims to better understand how these established patterns were altered by the pandemic in Belgrade, adding depth to the existing body of literature on the subject.

Lastly, we also recognize the limitations inherent in observational studies, particularly in their inability to establish causal relationships. While our study identifies associations between COVID-19 and excess mortality, further research, particularly prospective studies, would be necessary to more definitively address causal mechanisms.

Also, while linear regression was used to analyze trends in mortality, we acknowledge that Poisson regression may also be appropriate for rate data. Our study focused on overall mortality trends, and linear regression was deemed suitable given the aggregated nature of the data. Future studies could incorporate uncertainty and alternative modeling approaches to improve rigor.

### 4.16. Strengths of the Study

This study offers several key strengths that contribute to its robustness and value. First, the use of detailed mortality data from official public health sources in Belgrade from March 2020 to May 2023 provides a comprehensive view of mortality trends during the COVID-19 pandemic. Second, the incorporation of historical mortality data dating back to 1995 enhances the robustness and reliability of the expected mortality estimates, ensuring that conclusions are drawn from a well-rounded understanding of historical mortality patterns, seasonal effects, and long-term health trends. Furthermore, the study’s focus on excess mortality and the use of proportional excess mortality as a key measure allow for a nuanced assessment of the pandemic’s impact on public health. The combination of linear regression with seasonal adjustments also strengthens the analysis by accounting for seasonal fluctuations in mortality, offering a clear picture of how COVID-19 exacerbated mortality during specific periods, especially the winter months. Finally, the application of rigorous statistical methodologies and the careful consideration of local context make this study a valuable contribution to the growing body of knowledge on pandemic-related mortality trends.

### 4.17. Implications for Policy, Intervention Strategies, and Pandemic Preparedness

The findings of this study underscore the significant impact of the COVID-19 pandemic on mortality patterns in Belgrade, Serbia, highlighting seasonal fluctuations and excess mortality during the winter months. In addition to the clinical and epidemiological insights, these findings have important social and political implications that can inform future public health policies and intervention strategies. Firstly, the seasonal mortality trends observed in this study point to the urgent need for a comprehensive approach to public health preparedness, particularly during the winter months. Given that respiratory diseases, such as COVID-19 and influenza, tend to increase during this period, strengthening healthcare systems to cope with seasonal surges is essential. This could involve improving healthcare infrastructure, increasing access to medical resources, and ensuring adequate staffing during peak seasons. Additionally, policies aimed at promoting early detection and prevention, such as vaccination campaigns and public health awareness programs, would help mitigate the impact of respiratory illnesses during these vulnerable months. Furthermore, the mental health consequences of the pandemic, as highlighted in our study, suggest the need for long-term support systems. The societal and economic impacts of the pandemic, particularly in the context of isolation and economic instability, have exacerbated mental health issues. Policymakers should prioritize the development of mental health interventions, such as community-based programs, counseling services, and financial support for affected individuals. These efforts would not only alleviate the burden on healthcare systems but also help address the broader societal consequences of the pandemic. Finally, this study emphasizes the importance of strengthening pandemic preparedness for future public health crises. Policymakers should invest in robust surveillance systems, data collection frameworks, and predictive modeling techniques to better anticipate and respond to health threats. Moreover, fostering international collaboration in research and data sharing would enhance global responses to pandemics, ensuring that lessons learned from COVID-19 can be effectively applied in future situations. In summary, his study’s findings provide a valuable foundation for developing targeted interventions and policies that can reduce seasonal mortality, improve mental health outcomes, and enhance preparedness for future pandemics, ultimately leading to more resilient public health systems.

### 4.18. Future Research Directions

While this study focused on overall mortality patterns, it is important to acknowledge that further research is needed to explore the differential impact of the COVID-19 pandemic on various demographic groups. As our study was designed with a broad scope to analyze general mortality trends, it did not have the capacity to examine specific subgroups in detail. However, future studies should certainly address critical aspects such as age groups, sex differences, and the presence of comorbidities, as these factors are central to understanding the full spectrum of the pandemic’s impact on public health. Age groups, for example, may experience varying mortality rates, with older individuals being more vulnerable to severe outcomes, particularly during a health crisis like COVID-19. Similarly, gender differences in health outcomes, as well as the influence of pre-existing conditions such as cardiovascular diseases, diabetes, and respiratory illnesses, could provide valuable insights into how certain populations were disproportionately affected. These demographic and health-related factors should be considered in future studies to capture a more nuanced understanding of pandemic mortality. By stratifying mortality data and exploring these aspects, researchers will be able to identify the most vulnerable groups and better inform targeted interventions. Additionally, understanding how different demographic groups fared during the pandemic will allow policymakers to design more effective public health responses, ensuring that healthcare resources are allocated where they are needed most. Given the increasing importance of precision medicine and tailored interventions, such research is essential in mitigating the impacts of future public health emergencies. While our study lays the groundwork for understanding overall mortality patterns, future investigations that incorporate detailed data on age, sex, and comorbidities will offer more comprehensive insights and help refine health policies and preparedness strategies for subsequent health crises.

## 5. Conclusions

In conclusion, this study analyzed mortality rates and excess mortality in Belgrade from March 2020 to May 2023, with a focus on the COVID-19 pandemic’s impact on overall mortality. Our analysis revealed significant shifts in mortality patterns, notably the exacerbation of winter mortality during the pandemic. We found that the winter months saw a notable increase in excess deaths, particularly among vulnerable populations such as the elderly and individuals with comorbidities. These groups faced disproportionately high mortality, which was further exacerbated by COVID-19, thus highlighting the interaction between seasonal mortality patterns and the pandemic.

Specifically, we observed that comorbid conditions, including cardiovascular diseases, diabetes, and respiratory conditions, significantly contributed to higher mortality during both the winter season and the COVID-19 crisis. This underscores the critical need for focused public health interventions that address both the seasonal risks and the underlying comorbidities, which make certain populations more vulnerable during health crises.

Additionally, the study emphasizes the role of delayed medical treatments, disruptions in healthcare access, and the mental health consequences of grief, which collectively contributed to the excess mortality observed during the pandemic. These findings further reinforce the importance of mental health support and bereavement services, which should be integral components of any public health response during future pandemics.

Overall, we conclude that future health policies must focus on improving resilience within the healthcare system, enhancing mental health services, and ensuring equitable access to care for vulnerable groups, particularly during the winter months when mortality rates traditionally rise. Further research is necessary to explore the complex relationship between comorbidities, seasonal mortality, and the broader societal factors that contribute to higher death rates in order to develop more targeted, effective public health strategies.

## Figures and Tables

**Table 1:** Overview of the key aspects, findings, and implications.

Topic	Key Findings and Implications
COVID-19 Impact on Mortality Rates	COVID-19 became a leading cause of death globally and in Serbia, particularly among the elderly with pre-existing comorbidities. Mortality rates started increasing in late 2020.
Underreporting and Indirect Mortality	Official COVID-19 death counts likely underestimate actual deaths, with some estimates suggesting a 50% higher death toll due to underreporting, misclassification, and indirect deaths caused by healthcare system strain.
Excess Mortality and Broader Impacts	“Excess deaths” reflect the true mortality impact, including indirect deaths. COVID-19 became the second leading cause of death in Belgrade in 2020.
Vulnerability of Middle-Aged Individuals	Middle-aged men with comorbidities were at increased risk of COVID-19-related mortality, which refines prior research focusing mostly on older adults.
Non-Clinical Factors Influencing Mortality	Socioeconomic factors, healthcare access, and public health policies influenced mortality. Mental health issues, economic instability, and delayed treatments contributed to higher mortality.
Seasonal Mortality and Psychological Consequences	Seasonal mortality in winter increased, compounded by COVID-19. Bereavement and grief led to negative mental health outcomes, including depressive symptoms and reduced quality of life.
Socio-Medical Perspective	Public health and clinical measures, such as timely interventions and adherence to preventive measures, are crucial for addressing both direct and indirect mortality.
Insights on COVID-19 Mortality and Public Health	Excess deaths during COVID-19 reflect both direct and indirect consequences, highlighting the need for effective public health interventions and timely healthcare measures.
Relevance for Local and Global Setting	The pandemic worsened winter mortality, revealing vulnerabilities in public health systems and underscoring the importance of addressing both medical and social determinants of health.
Public Health Implications	The pandemic emphasizes the need for resilient public health systems that address both seasonal mortality fluctuations and the broader social determinants of health.
Clinical Implications	COVID-19 research has influenced global healthcare responses, focusing on prevention (mask-wearing, social distancing) and long-term effects such as respiratory and cardiovascular issues.

## Data Availability

Data for the study were sourced from two key institutions: the Republic Institute for Public Health of Serbia, which provided morbidity statistics, and the Republic Institute for Informatics and Statistics, from which mortality data were obtained. The combined dataset from these sources ensured a basis for the analysis.

## Abbreviations

COVID-19	Coronavirus disease 2019
SARS-CoV-2	Severe acute respiratory syndrome coronavirus 2
p-Excess MT	proportional excess mortality rate
YLLs	years of life lost
